# RNA Sequencing Analysis and Atrial Natriuretic Peptide Production in Patients with Dilated and Ischemic Cardiomyopathy

**DOI:** 10.1371/journal.pone.0090157

**Published:** 2014-03-05

**Authors:** Estefanía Tarazón, Esther Roselló-Lletí, Miguel Rivera, Ana Ortega, Maria Micaela Molina-Navarro, Juan Carlos Triviño, Francisca Lago, José Ramón González-Juanatey, Placido Orosa, José Anastasio Montero, Antonio Salvador, Manuel Portolés

**Affiliations:** 1 Cardiocirculatory Unit, Instituto de Investigación Sanitaria Hospital Universitario La Fe, Valencia, Spain; 2 Sistemas Genómicos, Valencia, Spain; 3 Cellular and Molecular Cardiology Research Unit, Department of Cardiology and Institute of Biomedical Research, University Clinical Hospital, Santiago de Compostela, Spain; 4 Cardiology Unit, Hospital San Francesc de Borja, Gandía, Spain; 5 Servicio de Cirugía Cardiovascular, Hospital Universitario La Fe, Valencia, Spain; 6 Heart Failure and Transplantation Unit, Cardiology Department, Hospital Universitario La Fe, Valencia, Spain; University of Otago, New Zealand

## Abstract

**Background:**

The atrium is the major site of ANP synthesis, which has been said to increase in heart failure as a result of increased production in the left ventricular (LV) myocardium. This is a key issue related to its diagnostic and prognostic capabilities. We aimed to evaluate protein levels of proANP and ANP and the enzymes that cleave the natriuretic peptides, corin and furin, in the LV tissue of heart transplant patients with dilated (DCM) and ischemic (ICM) cardiomyopathy compared with control donors (CNT). We also evaluate mRNA levels of ANP gene (*NPPA*) by RNA sequencing in the same tissue.

**Methods and Results:**

Seventy-three human LV tissue samples from ICM (n = 30) and DCM (n = 33) patients and CNT (n = 10) were analyzed by Western blot and RNA sequencing. Comparing protein levels according to etiology, neither DCM nor ICM showed levels of proANP or ANP different from those of CNT. However, *NPPA* was increased in both groups compared to CNT (DCM 32 fold, p<0.0001; ICM 10 fold, p<0.0001). Corin (but not furin) was elevated in the ICM group compared to CNT (112±24 *vs.* 100±7, p<0.05), and its level was inversely related with LV ejection fraction (LVEF) (r = −0.399, p<0.05).

**Conclusions:**

Patients present with elevated levels of *NPPA* but not of proANP or ANP proteins in LV tissue, which may be due to posttranscripcional regulation of *NPPA* or different pathways for ANP secretion between the atrium and ventricle. Moreover, there are differences between DCM and ICM in corin levels, indicating that a different molecular mechanism may exist that converge in this syndrome. Further, LV concentration of corin is inversely related to LVEF in ICM.

## Introduction

Heart failure (HF) is caused by any condition that reduces the efficiency of the myocardium through damage or overloading. Natriuretic peptides (NP) are a family of peptides that cause effects such as diuresis, natriuresis, vasodilation, and inhibition of aldosterone synthesis and renin secretion; playing an important role in regulating blood pressure and blood volume [1]. Our group has extensively studied the NP in plasma, urine and tissue, and their value in monitoring patients with hypertension and HF [2]–[6]. NP concentrations in plasma are routinely used in clinical medicine to aid in the diagnosis, prognosis, and determination of the severity of HF: specifically atrial natriuretic peptide (ANP) and brain natriuretic peptide (BNP) [7]–[10].

ANP and BNP are produced as a prohormones, proANP and proBNP, which are cleaved by 2 endoproteases (corin and furin) to form active ANP and BNP and inactive N-terminal molecules [11]. Corin was identified as the proANP convertase [12], although it also cleaves proBNP [13]; however, in this case it is less specific to sequence and less efficient. Other enzymes, such as furin, are responsible for processing proBNP more efficiently [13]. Recently, it has been shown that plasma levels of corin are lower in patients with HF compared to controls, relating with the severity of HF. However, no differences were found in patients with acute myocardial infarction [14]. Moreover, another study in a canine model of HF found higher corin and furin levels in HF left atrium but not in HF left ventricle compared with controls [15].

ANP is synthesized and secreted in the atria under normal conditions and by the ventricular myocardium during fetal development, hypertrophy, or HF [16]. Since ANP plasma levels are elevated in HF, it has been speculated that the extra ANP may originate in the left ventricular (LV) tissue of these patients [16]. This is a key issue related to its diagnostic and prognostic capabilities. Nevertheless, studies of its tissue levels are scant, most of them measuring mRNA levels. We hypothesized that the increased ANP levels could be produced in the LV tissue of patients with dilated (DCM) and ischemic (ICM) cardiomyopathy. Thus our objective was to evaluate for the first time mRNA levels of the ANP gene (*NPPA*) by RNA sequencing (RNA-seq) in the LV tissue of HF patients undergoing heart transplantation compared with healthy controls. Furthermore, we measured protein levels of ANP and proANP, and its related enzymes corin and furin, in the same LV tissue.

## Methods

### Ethics Statement

All patients gave their written informed consent to participate in the study. The project was approved by the Biomedical Investigation Ethics Committee of “La Fe” University Hospital of Valencia, Spain. The investigation was conducted in accordance with the guidelines of the Declaration of Helsinki [17].

### Source of tissue

LV tissue samples were obtained from 73 subjects: 63 patients with HF (33 with non-ischemic DCM and 30 with ICM) undergoing cardiac transplantation and 10 control (CNT) samples from non-diseased donor hearts [18]. All donors had normal LV function (>50) determined by echocardiography and no history of myocardial disease or active infection at the time of transplantation. The CNT hearts were considered for cardiac transplantation but were subsequently deemed unsuitable because of either blood type or size incompatibility. The cause of death was cerebrovascular (68%) or motor vehicle accident (32%).

The clinical characteristics of patients are shown in Table 1. Clinical history, ECG, hemodynamic studies, Doppler echocardiography, and coronary angiography data were available. No patients had signs of primary valvular disease. All patients had been classified according to the New York Heart Association (NYHA) functional criteria and were receiving medical treatment according to the guidelines of the European Society of Cardiology [19].

**Table 1:** Clinical characteristics of HF patients.

	DCM (n = 33)	ICM (n = 30)
**Age (years)**	49±13	54±7*
**Gender male (%)**	77	100
**NYHA class**	3.3±0.5	3.5±0.4
**BMI (kg/m^2^)**	25±6	28±4
**Hemoglobin (mg/mL)**	13±2	14±2
**Hematocrit (%)**	40±6	41±5
**Total Cholesterol (mg/dL)**	143±41	186±51***
**Prior hypertension (%)**	27	46
**Prior diabetes mellitus (%)**	16	54**
**Prior smoking (%)**	67	86
**EF (%)**	21±8	24±6
**FS (%)**	11±4	14±4
**LVESD (mm)**	65±11	55±8***
**LVEDD (mm)**	74±11	63±8***
**LVMI (g/cm^2^)**	204±64	138±33***
**Duration of disease (months)**	72±57	44±42
**Treatment (%)**		
**Angiotensin-converting enzyme inhibitors**	83	80
**Angiotensin II receptor antagonists**	10	15
**β-blokers**	63	30*
**Aldosterone antagonists**	88	70
**Digoxin**	67	45
**Antidiabetics**	17	50*
**Diuretics**	92	75
**Statins**	25	80***
**Antithrombotics**	63	85
**Antiarrhythmics**	21	15

BMI, body mass index; DCM, dilated cardiomyopathy; Duration of disease from the diagnosis of heart failure until heart transplant; EF, ejection fraction: FS, fractional shortening; NYHA, New York Heart Association; LVEDD, left ventricular end-diastolic diameter; LVESD, left ventricular end-systolic diameter; LVMI, left ventricular mass index.

*p<0.05;

**p<0.01;

***p<0.001.

LV samples were taken from a portion proximal to the apex of the left ventricle. The DCM, ICM and CNT samples were stored in 0.9% NaCl at 4°C for a mean time of 4.4±3 h after the loss of coronary circulation.

### Homogenization of samples and protein determination

Twenty-five milligrams of frozen left ventricle were homogenized in a total protein extraction buffer (2% SDS, 10 mM EDTA, 6 mM Tris–HCl, pH 7.4) with protease inhibitors (25 µg/mL aprotinin and 10 µg/mL leupeptin) in a FastPrep-24 homogenizer (MP Biomedicals, USA) with specifically designed Lysing Matrix D tubes. The homogenates were centrifuged, and the supernatant was aliquoted. The protein content of the aliquot was determined by Peterson's Modification [20] of the Lowry method using bovine serum albumin (BSA) as standard.

### Polyacrylamide gel electrophoresis and Western blot analysis

Forty-five micrograms of each homogenate were separated by Bis-Tris Midi gel electrophoresis with 4–12% polyacrylamide in separate gels for proANP, ANP, corin, and 10% for furin. For the Western blot, the proteins were transferred from the gel to a PVDF membrane, using an iBlot Dry Blotting System (Invitrogen, UK). The membrane was blocked for the entire night at 4°C with 1% BSA in Tris-buffer solution containing 0.05% Tween 20. After blocking, membranes were incubated for 2 h with the primary antibody in the same buffer. The primary detection antibodies used were: a mouse monoclonal anti-human IgG against proANP and ANP (1∶5000 dilution; MD-17-0027) (RayBiotech, Norcross, GA, USA), and a mouse monoclonal anti-human IgG against corin (1∶100 dilution; ab56983) and a rabbit polyclonal anti-human IgG against furin (1∶1000 dilution; ab28548) (Abcam, Cambridge, UK). A mouse monoclonal anti-human IgG against GAPDH antibody (1∶10000 dilution; ab9484; Santa Cruz Biotechnology, CA, USA) was used as the loading control for each of the blots.

The bands were visualized using an acid phosphatase-conjugated secondary antibody and nitro blue tetrazolium/5-bromo-4-chloro-3-indolyl phosphate (NBT/BCIP, Sigma-Aldrich, St. Louis, MO, USA) substrate system. Finally, bands were digitalized using an image analyzer (DNR Bio-Imaging Systems, Israel) and quantified by the Gel Capture (v. 4.30) and the GelQuant Pro (v. 12.2) programs.

### RNA extraction

Heart samples were homogenized with TRIzol agent in a TissueLysser LT (Qiagen, UK). All RNA extractions were performed using a PureLink Kit according to the manufacturer's instructions (Ambion Life Technologies, CA). RNA was quantified using a NanoDrop1000 spectrophotometer (Thermo Fisher Scientific, UK) and the purity and integrity of RNA samples was measured using an Agilent 2100 Bioanalyzer with the RNA 6000 Nano LabChip kit (Agilent Technologies, Spain). All samples displayed a 260/280 ratio greater than 2.0 and RNA integrity numbers ≥9.

### RNA-seq

PolyA-RNA was isolated form 25 micrograms of total RNA using the MicroPoly(A) Purist kit (Ambion, USA). Total PolyA-RNA samples were used to generate whole transcriptome libraries for sequencing on the SOLiD 5500XL platform, following the manufacturer's recommendation (Life Technologies, CA). No RNA-spike in controls was used. Amplified cDNA quality was analyzed by the Bioanalyzer 2100 DNA 1000 kit (Agilent Technologies, Spain) and quantified using the Qubit 2.0 Fluorometer (Invitrogen, UK). The whole transcriptome libraries were used for making SOLiD templated beads following the SOLiD Templated Bead Preparation guide. Bead quality was estimated based on WFA (workflow analysis) parameters. The samples were sequenced using the 50625 paired-end protocol, generating 75 nt+35 nt (Paired-End)+5 nt (Barcode) sequences. Quality data were measured using software SETS parameters (SOLiD Experimental Tracking System).

### Computational analysis of RNA-seq data

The initial whole transcriptome paired-end reads obtained from sequencing were mapped against the latest version of the human genome (version GRchr37/hg19) using the Life Technologies mapping algorithm (http://www.lifetechnologies.com/), version 1.3. It was using the standard Bioscope parameters of version 1.3, in paired ends and whole transcriptome analysis. For both reads, forwards and revers, the seed was the first 25 nucleotides with a maximum of 2 mismatches allows, tables S1 and S2 describe the main statistical parameters of mapping step of analysis. The aligned records were reported in BAM/SAM format [21]. Bad quality reads (Phred score <10) were eliminated using Picard Tools software, version 1.83 [22].

Subsequently, isoforms and gene prediction were estimated using the cufflinks method [23] and the expression levels were calculated using the htseq software, version 0.5.4p3 [24], this method eliminate the multimapped reads, only the unique reads are consider for gene expression estimation. Edge method, version 3.2.4, was applied for differential expression analysis between conditions [25]. This method rely on different normalize process based in depth global samples, CG composition and length of genes. In the differential expression process, this method relies on a Poisson model to estimate the variance of the RNA-seq data for differential expressions. The data discussed in this publication have been deposited in NCBI's Gene Expression Omnibus and are accessible through GEO Series accession number GSE55296 (http://www.ncbi.nlm.nih.gov/geo/query/acc.cgi?acc=GSE55296).

### Statistical methods

Data are presented as mean value ± standard deviation for continuous variables and as percentage for discrete variables. The Kolmogorov-Smirnov test was used to analyze the distribution of the variables. Comparisons of clinical characteristics were achieved using Student's t-test for continuous variables and Fisher's exact test for discrete variables. Comparisons of tissue levels of proteins by Western blot were performed using Student's t-test, and Pearson's correlation coefficients were calculated to determine the relationships among levels of proteins and clinical characteristics. Significance was assumed as p<0.05. All statistical analyses were performed using SPSS software v. 20 for Windows (IBM SPSS Inc., Chicago. IL, USA).

## Results

### Clinical characteristics of patients

We analyzed 73 human hearts; heart tissue was explanted from 63 patients undergoing cardiac transplantation after being diagnosed with non-ischemic DCM (n = 33) or ICM (n = 30) cardiomyopathy, and CNT samples were obtained from 10 non-diseased donor hearts.

Most of the patients were men (87%) and their mean age was 51±11 years. The patients had an NYHA functional classification of III–IV and had previously been diagnosed with significant comorbidities, including hypertension (34%), hypercholesterolemia (13%), and diabetes mellitus (32%). The CNT group mainly comprised men (73%) and had a mean age of 49±19 years. Table 1 shows the clinical characteristics of the patients according to etiology. Compared to the DCM group, the ICM group had significantly greater age (p<0.05), higher cholesterol levels (p<0.001), and a higher rate of prior diabetes mellitus (p<0.001), whereas DCM patients had larger values for LV end-systolic diameter (LVESD) (p<0.001), LV end-diastolic diameter (LVEDD) (p<0.001), and LV mass index (p<0.001). Moreover, statistically significant changes were found in β-blockers (p<0.05), antidiabetics (p<0.05) and statins (p<0.001) according to etiology of HF.

### Effects of HF on protein and mRNA levels

**Figure 1:**
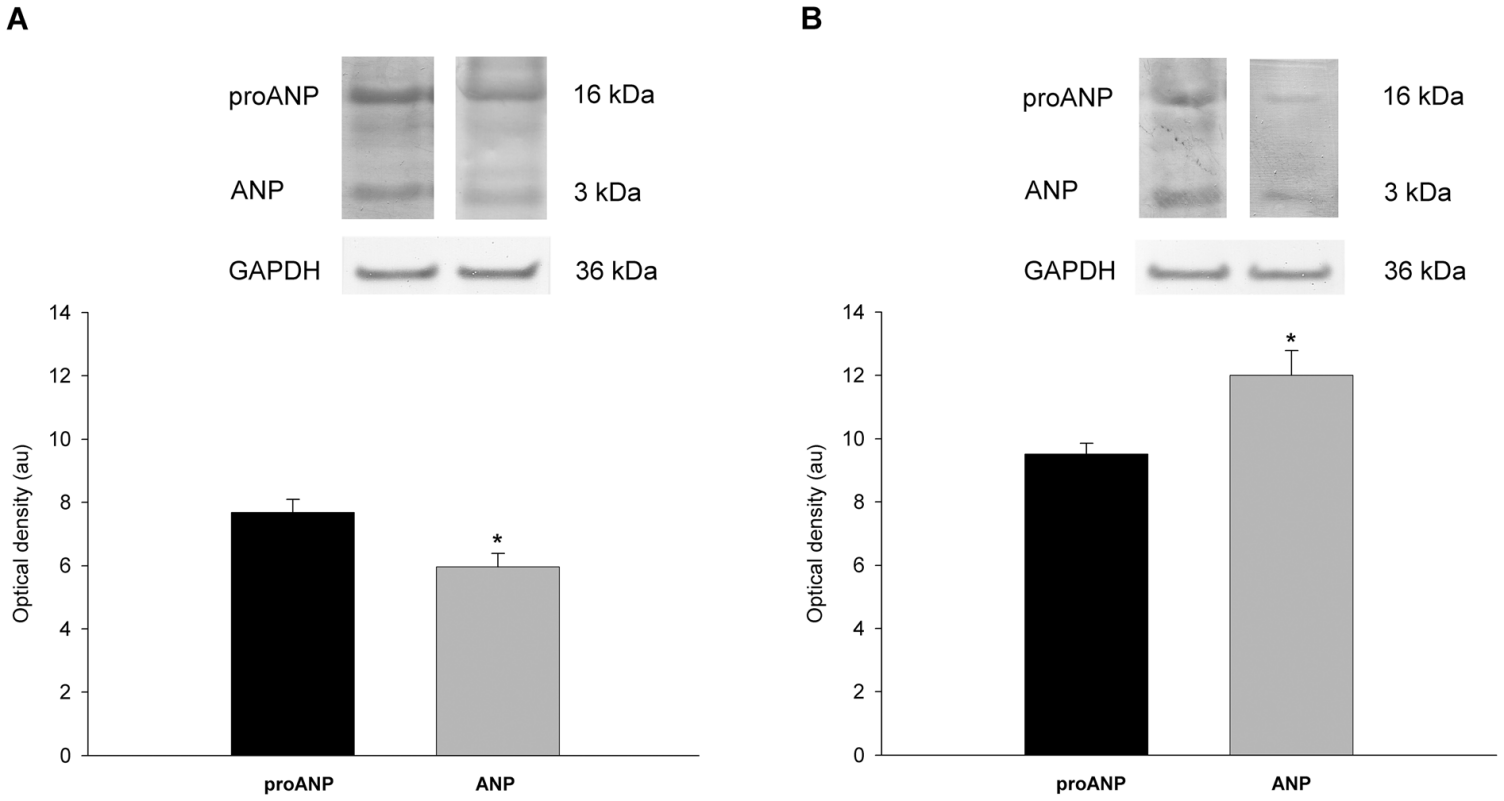
Atrial natriuretic peptide levels in patients with heart failure. The data are expressed in arbitrary units (optical density) as the mean ± SEM. Pro-atrial natriuretic peptide (proANP) versus atrial natriuretic peptide (ANP) in dilated (A) and ischemic (B) cardiomyopathy. *p<0.01.

To analyze the pathophysiology of NP and enzymes in cardiac tissue, we used Western blot techniques to determine the protein levels of the immunoreactive forms of the NP (proANP, and ANP) and the endoproteases (corin and furin) in dilated and ischemic human hearts. When we compared NP levels within the DCM group, we observed that tissue proANP levels were higher than ANP levels (29%, p<0.01). In contrast, within the ICM group, tissue proANP levels were lower than ANP levels (26%, p<0.01) (Figure 1).

When we compared the protein levels in HF patients and CNT, proANP and ANP did not show any significant differences (102±21 *vs.* 100±15, p = 0.728; 89±25 *vs.* 100±17, p = 0.160, respectively). Similarly, there were no significant differences in corin and furin between the HF patients and the CNT group: 104±30 *vs.* 100±7, p = 0.334 for corin; and 101±46 *vs.* 100±33, p = 0.971 for furin.

**Figure 2:**
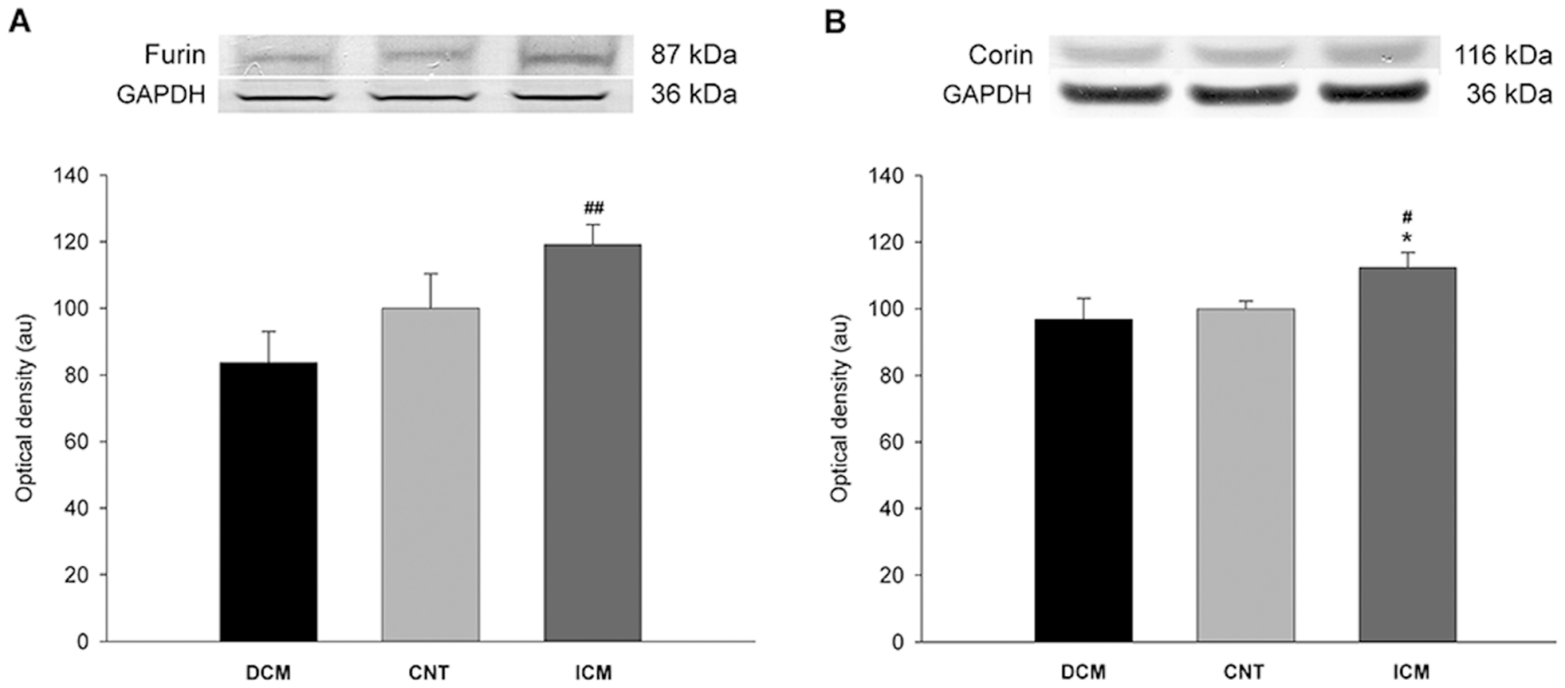
Protein expression levels of endoproteases in the dilated (DCM) and ischemic (ICM) cardiomyopathy, and control (CNT) groups. A, furin; B, corin. The values from the CNT group were set to 100. The data are expressed as mean ± SEM in arbitrary units (optical density) of 2 independent experiments. *p<0.05 *vs.* the CNT group; ^#^p<0.05, ^##^p<0.01 DCM *vs.* the ICM group.

When protein levels were compared according to the etiology of HF, proANP and ANP levels did not show any significant difference from controls (94±15 *vs.* 100±15, p = 0.258; and 92±17 *vs.* 100±17, p = 0.194, respectively, for the DCM group; 112±22 *vs.* 100±15, p = 0.122; and 85±31 *vs.* 100±17, p = 0.151, respectively, for the ICM group). The same applied to furin levels (84±51 *vs.* 100±33, p = 0.353; 119±32 *vs.* 100±33, p = 0.113) for DCM and ICM, respectively, compared to CNT; however, we noted that there was an increase in furin levels in ICM respect to the DCM group (119±32 *vs.* 84±51, p<0.01) (Figure 2A). Corin showed higher levels in ICM hearts (112±24 *vs.* 100±7, p<0.05), but not in DCM hearts (97±33 *vs.* 100±7, p = 0.634) compared to CNT; besides the ICM group also showed higher levels compared to DCM group (112±24 *vs.* 97±33, p<0.05) (Figure 2B).

**Figure 3:**
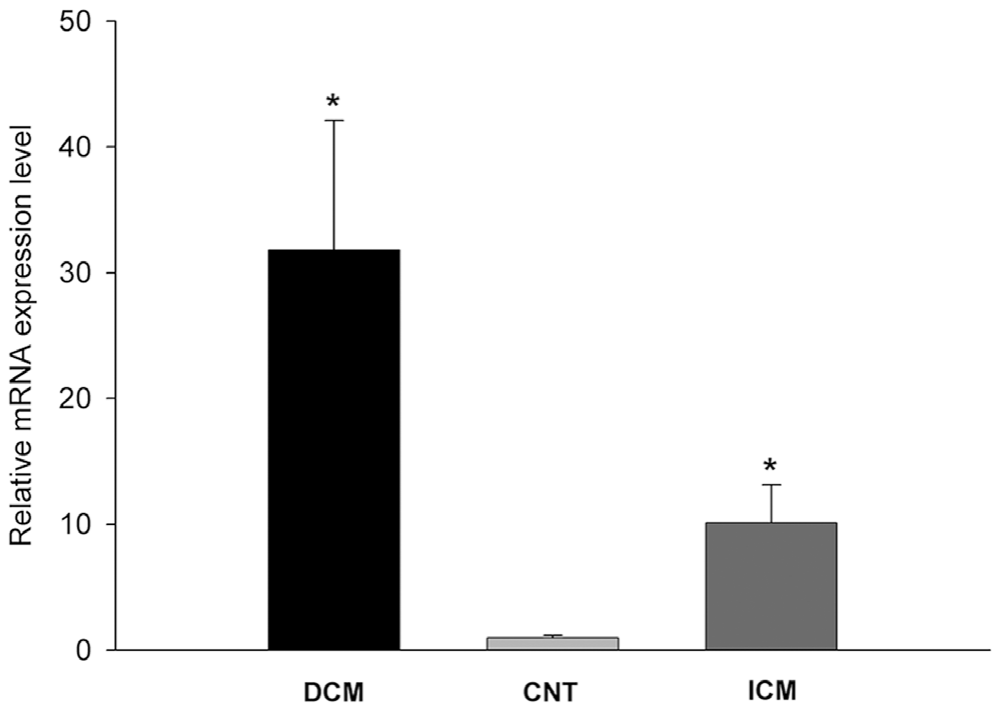
Levels of proANP mRNA expression determined by RNA-seq. The values from the CNT group were set to 1. The data are expressed as mean ± SEM in mRNA relative expression. DCM, dilated cardiomyopathy; ICM, ischemic cardiomyopathy; CNT, control. *p<0.0001.

The mRNA differences between patients and CNT were determined by RNA-seq. *NPPA* was increased in both the DCM (32 fold, p<0.0001) and ICM (10 fold, p<0.0001) groups, compared to the CNT group (Figure 3). In both groups, we found that only 1 of the 2 known *NPPA* gene isoforms was differentially expressed: ENST00000376476, 32 fold in DCM (p<0.0001) and 13 fold in ICM (p<0.0001).

### Relationships between proteins, mRNA and LV function

We further determined whether there was any relationship among the levels of each of the proteins and between mRNA and protein levels. We found that corin and proANP were related in both etiologies, DCM (r = 0.499, p<0.01) and ICM (r = 0.574, p<0.001) (Figure S1A and S1B). We also found a relevant relationship between 2 enzymes in both, DCM (r = 0.616, p<0.0001) and ICM (r = 0.502, p<0.01) groups (Figure S2).The proANP and ANP levels are interrelated, and we found a strong relationship between them (r = 0.547, p<0.001) in the DCM group. Finally, corin had a significant correlation with ANP (r = 0.455, p<0.05) in the ICM group (Figure S3).

Finally, we determined whether there was any relationship in protein and mRNA levels with changes in LV function. For 27 out of 30 samples the LV function parameters were completely available in ICM. We found a significant inverse correlation between corin levels and LV ejection fraction (EF) in ICM (r = −0.399, p<0.05) (Figure S4).

## Discussion

There are few studies of ANP in LV tissue; most of them explored the changes at the mRNA level [26]–[27]; while some reported important limitations [16]. Since ANP plasma levels are elevated in HF, it has been suggested that the ANP could originate in the LV tissue of these patients [16], [26]–[29]. We hypothesized that ANP levels could be augmented by ANP produced by LV tissue in patients with DCM and ICM. Therefore, our objective was to evaluate for the first time the *NPPA* levels by RNA-seq in the LV tissue of HF patients undergoing heart transplantation, compared with healthy controls. In addition, we measured protein levels of ANP and proANP, and its related enzymes corin and furin, in the same LV tissue.

This study offers several insights into the tissue expression of ANP forms in human hearts. We determined the proANP and ANP expression by Western blot techniques and we were able to find both molecular forms in the LV tissue of all groups. Therefore, we concluded that ANP forms are present in adult LV tissue, although the highest levels are found in fetal stages and decline during the progress of gestation in developing hearts [16], [30]–[31]. We also found that the proANP form predominated over the ANP form in the DCM group, while the reverse was true in the ICM group. This was consistent with our finding that corin was elevated in the ICM group but not in the DCM group. It has been previously reported that changes in corin levels affect the levels of proANP and ANP in plasma [32]. Therefore, a greater amount of enzyme will cleave proANP, increasing ANP levels.

The literature includes reports of increases in proANP mRNA in the left ventricle, something we can also observe with RNA-seq experiments. This technique provides a far more precise measurement of levels of transcripts and their isoforms than microarray technology [33]. However, our observations indicate that there are no differences in the protein levels of proANP and ANP in the LV tissue of these 2 etiologies, compared with CNT. A recent study in an experimental model of HF shows similar ANP protein levels in LV when compared with normals [15]. It may be regulation at the mRNA level that prevents the RNA level changes being observed at the protein level. In this sense, Arora et al have recently discovered that microRNA-425 binds in an allele-specific manner to *NPPA*, acting as a negative regulator of the ANP production [34]. This mechanism or similar could be upregulated, producing the discrepancy between mRNA and protein levels. Indicating that, at least in the area of the left ventricular apex where we collect our samples, ANP production is not increased. Thus, the increase in these peptides observed in plasma could be due to synthesis in other areas of the myocardium. However, Bloch et al observed that ANP secretion differs between atrial and ventricular cardiomyocytes. Atrial cells store peptide in the secretory granules, whereas ventricular cells rapidly secrete the peptide following synthesis [31]. Based on this, we speculated that there could be an increase in ventricular ANP synthesis unobservable at the protein level due to the speed of secretion to plasma, thus contributing to the elevated peptide levels in HF. The production of ANP in the left ventricle would reveal that the increase in plasma levels should not only be due to the stretch of the atrial fibers but maybe to a potential ventricular stretching or focal ischemic injury.

Previous studies have established a correlation between the forms of plasma NP [33], [35]. In this regard, we found a strong association between proANP and ANP in the tissue of the DCM group. However, this correlation was not observed in the ICM group. Therefore, we believed that the higher corin levels change the relationship between these 2 peptides, which would be consistent with the correlation we found between ANP and corin in the ICM group. In addition, we found a strong relationship between proANP and corin in both etiologies but none with furin. This is in agreement with previous studies indicating that corin can process proANP and proBNP, although the latter less efficiently, whereas furin processes only proBNP [12]–[13].

LV function parameters are closely related to the ventricular remodeling that occurs in HF progression. A long-term remodeling process becomes detrimental leading to a progressive cardiac decompensation [36]. We determined whether there were any relationships between protein levels and the LV function parameters shown in Table 1. We found that corin was inversely related with LVEF only in the ICM group: in other words, higher levels of corin are linked with LV function impairment. This is in agreement with our data since corin has significantly altered levels in this group and not in DCM group.

From the clinical point of view, our findings allow us to better understand the pathophysiology of the ANP. Furthermore, it appears discarded that part of the higher ANP concentration in plasma in these patients could be due to an increased production of ANP in its left ventricular fibers, at least in the apical region.

A limitation of this study is the inherent variability of the samples, given their origin from human hearts and the medical treatments that the patients were undergoing. Furthermore, we chose DCM patients who did not report any family history of the disease. Our study is confined to the LV apex and therefore, we cannot exclude an increased production of ANP in other regions of the left ventricle. However, we want to emphasize the importance of having carried out this study in a significant number of samples from explanted human hearts from DCM and ICM patients undergoing cardiac transplantation. This has allowed us to extract the region of tissue that we wanted to analyze, which could not have been possible if the study had been made using biopsied tissues. However, we do not have atrial tissue of these patients in our collection of samples.

## Conclusion

In summary, this study shows that patients with dilated and ischemic cardiomyopathy present elevated expression of *NPPA* mRNA but not of proANP or ANP protein levels in human left ventricular apical tissue, which may be due to posttranscripcional regulation of *NPPA* or different pathways for ANP secretion between the atrium and ventricle. Moreover, we observed differences between the two etiologies in the tissue levels of corin, indicating that different molecular mechanism may exist that converge in this syndrome. Further, left ventricular levels of corin are inversely related to the ventricular ejection fraction of patients with ischemic cardiomyopathy.

## Supporting Information

Figure S1 **Correlation between pro-atrial natriuretic peptide and corin.** The scatter plots showed that pro-atrial natriuretic peptide (proANP) was correlated with corin in dilated cardiomyopathy (A), and in ischemic cardiomyopathy (B). Values were normalized to GAPDH and finally to the CNT group.
(TIF)

Figure S2 **Correlation of endoproteases.** The scatter plots showed the correlations of furin and corin in dilated cardiomyopathy (A) and ischemic cardiomyopathy (B). Values were normalized to GAPDH and finally to the CNT group.
(TIF)

Figure S3 **Correlation between atrial natriuretic peptide and corin in ischemic cardiomyopathy.** Values were normalized to GAPDH and finally to the CNT group.
(TIF)

Figure S4 **Correlation between corin and ejection fraction.** Values were normalized to GAPDH and finally to the CNT group.
(TIF)

Table S1 **Main statistical parameters of mapping step of analysis.**
(XLSX)

Table S2 **Percentage splicing reads of NPPA gene.**
(XLSX)
