# Supplementary figures and images for: RNA Sequencing Analysis and Atrial Natriuretic Peptide Production in Patients with Dilated and Ischemic Cardiomyopathy
Source: PLoS One. 2014 Mar 5;9(3):e90157. doi: 10.1371/journal.pone.0090157 (PMC3943898; doi:10.1371/journal.pone.0090157)

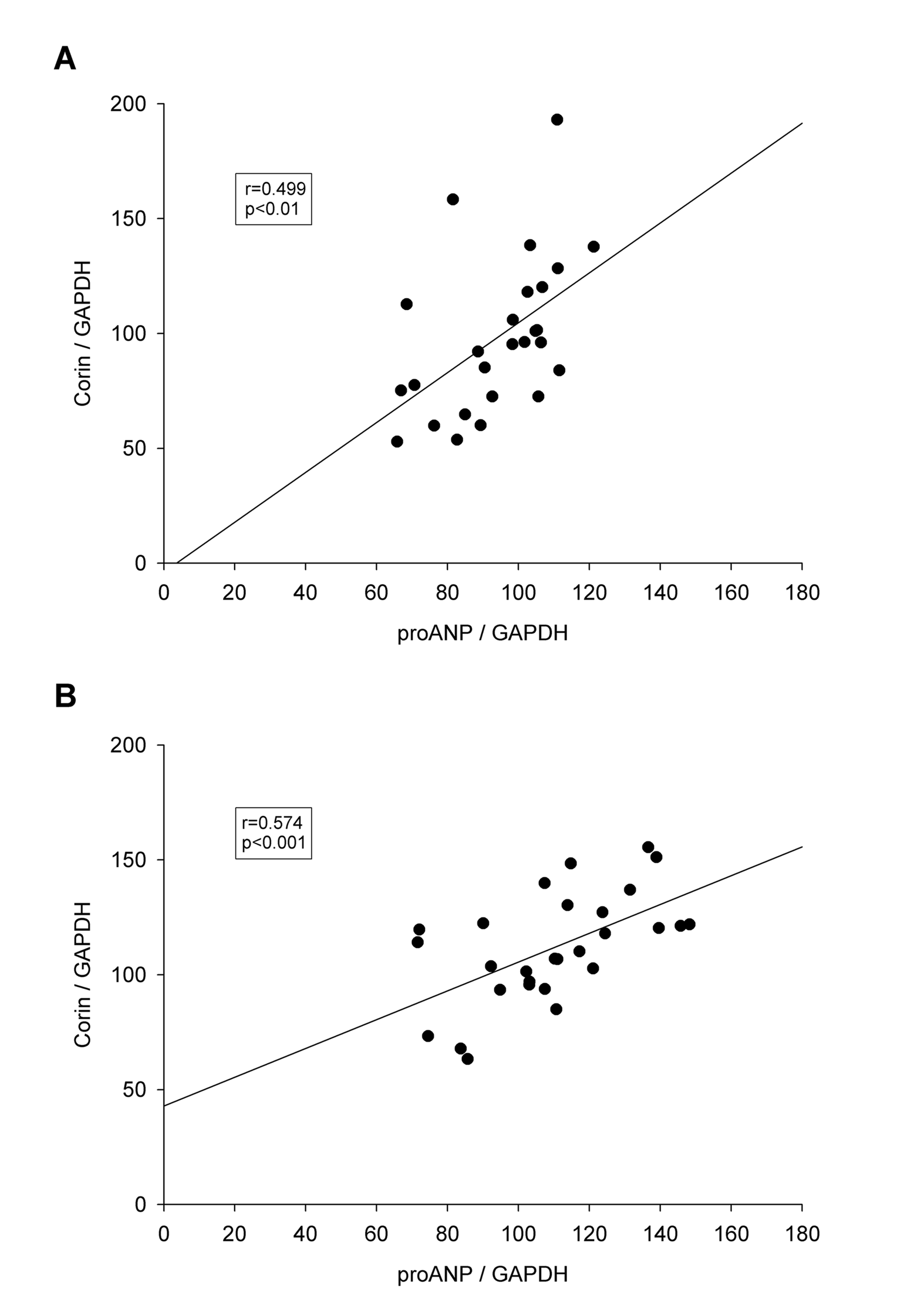

Supplement: Figure S1 — Correlation between pro-atrial natriuretic peptide and corin. The scatter plots showed that pro-atrial natriuretic peptide (proANP) was correlated with corin in dilated cardiomyopathy (A), and in ischemic cardiomyopathy (B). Values were normalized to GAPDH and finally to the CNT group. (TIF) [file pone.0090157.s001.tif]

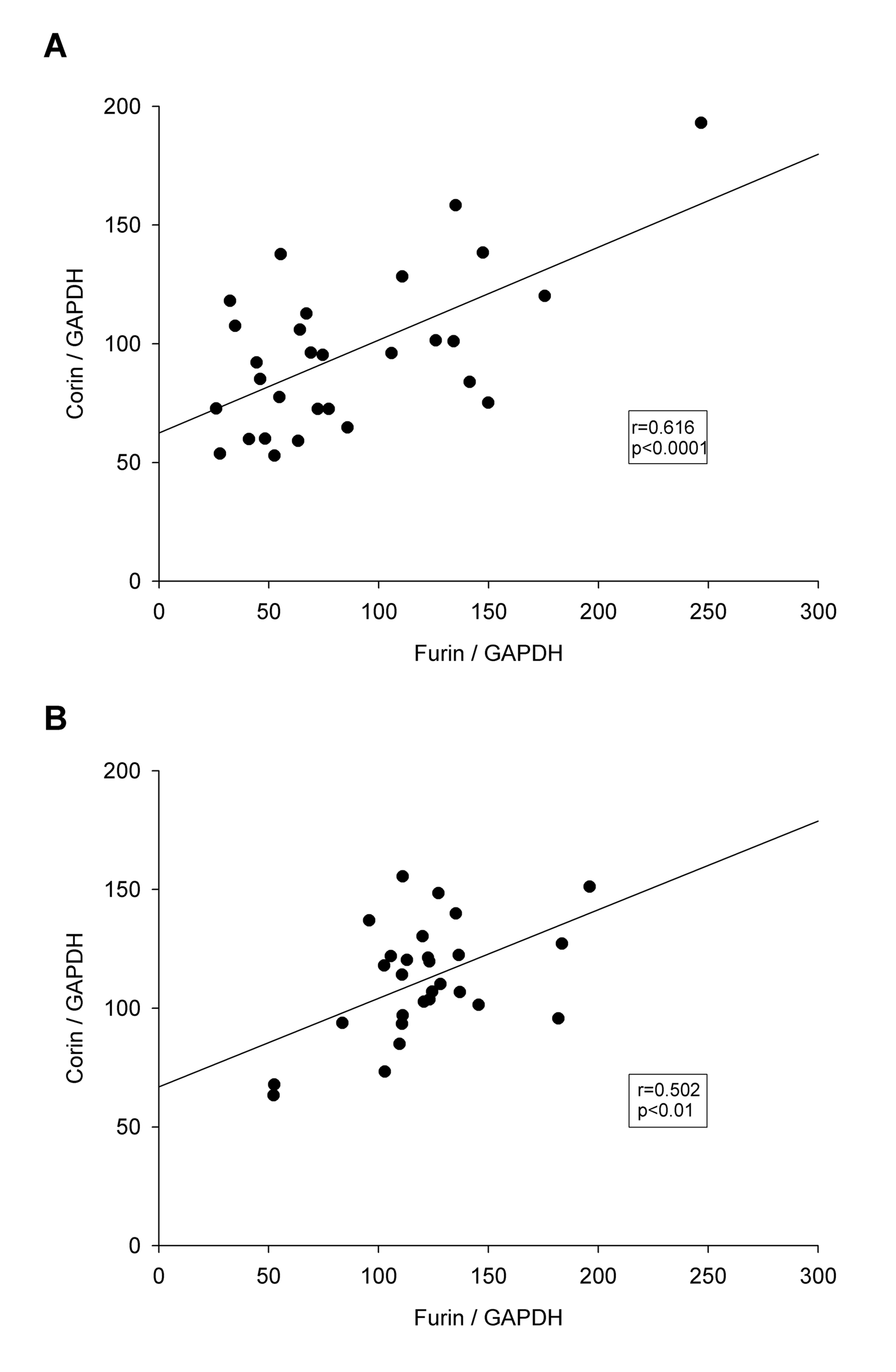

Supplement: Figure S2 — Correlation of endoproteases. The scatter plots showed the correlations of furin and corin in dilated cardiomyopathy (A) and ischemic cardiomyopathy (B). Values were normalized to GAPDH and finally to the CNT group. (TIF) [file pone.0090157.s002.tif]

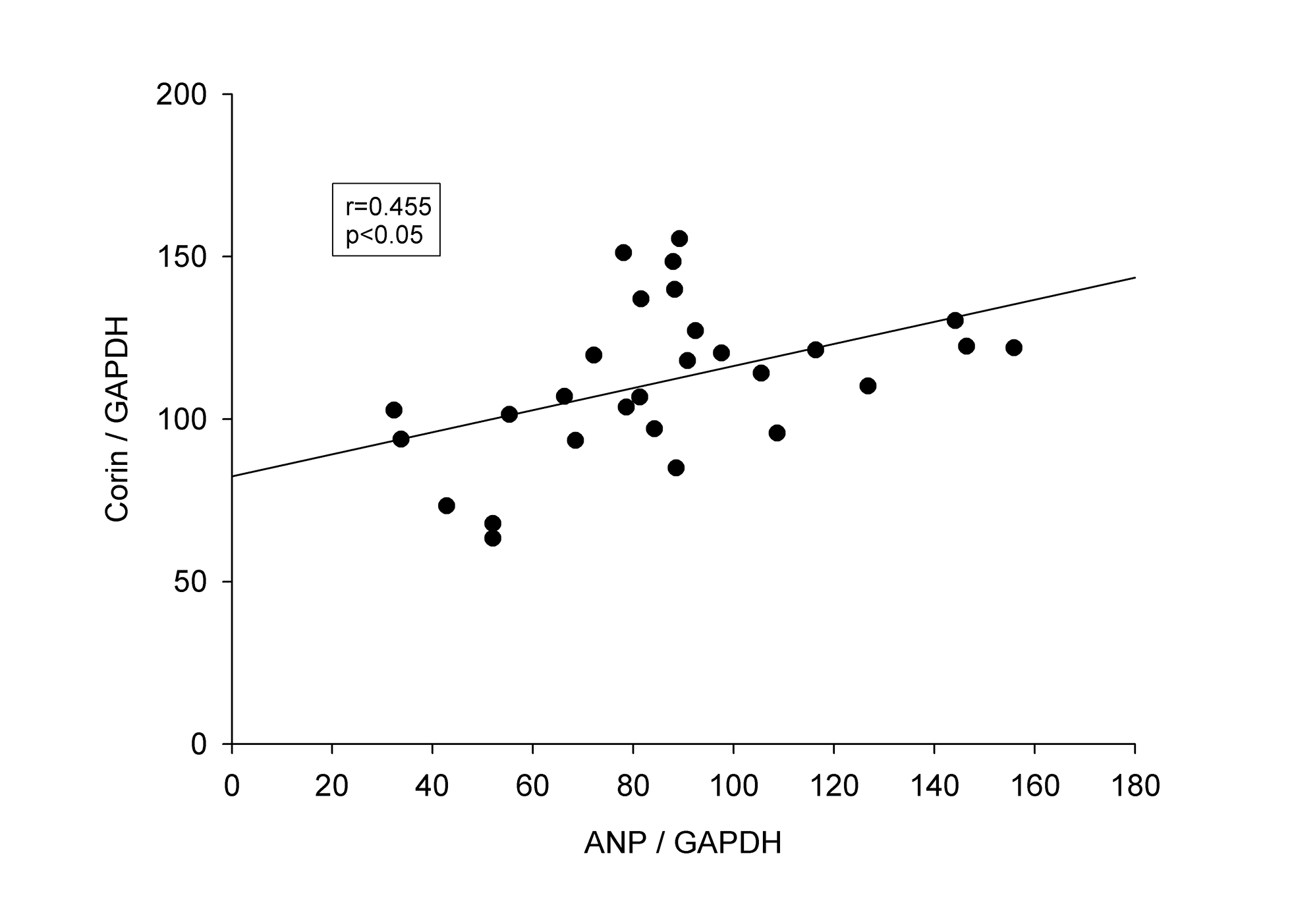

Supplement: Figure S3 — Correlation between atrial natriuretic peptide and corin in ischemic cardiomyopathy. Values were normalized to GAPDH and finally to the CNT group. (TIF) [file pone.0090157.s003.tif]

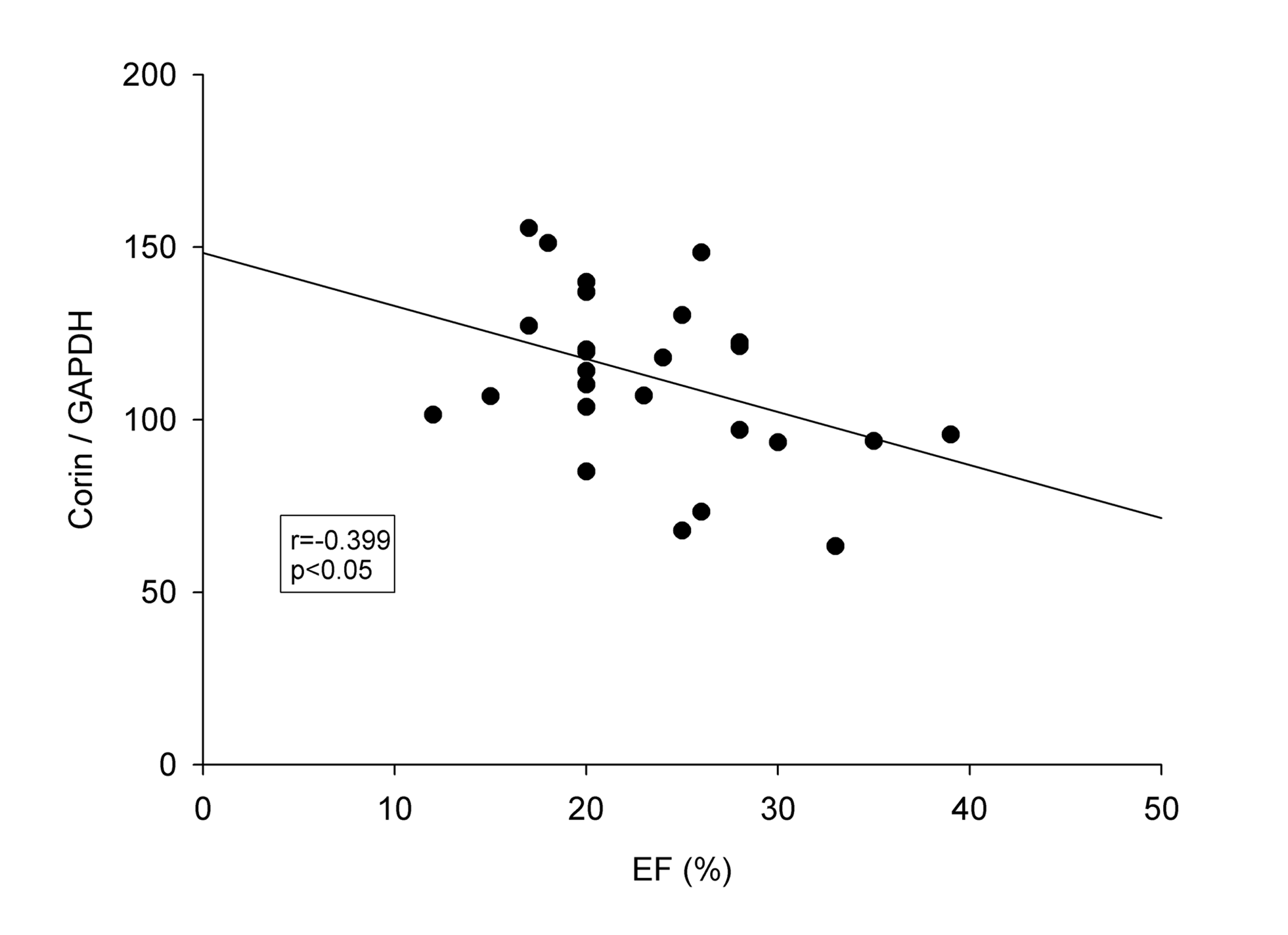

Supplement: Figure S4 — Correlation between corin and ejection fraction. Values were normalized to GAPDH and finally to the CNT group. (TIF) [file pone.0090157.s004.tif]
